# The Effect of Storage Condition and Duration on the Deterioration of Primed Rice Seeds

**DOI:** 10.3389/fpls.2018.00172

**Published:** 2018-02-13

**Authors:** Weiqin Wang, Aibin He, Shaobing Peng, Jianliang Huang, Kehui Cui, Lixiao Nie

**Affiliations:** ^1^MOA Key Laboratory of Crop Ecophysiology and Farming System in the Middle Reaches of the Yangtze River, College of Plant Science and Technology, Huazhong Agricultural University, Wuhan, China; ^2^Hubei Collaborative Innovation Center for Grain Industry, Yangtze University, Jingzhou, China

**Keywords:** seed priming, seed longevity, storage condition, starch metabolism, lipid peroxidation, antioxidant enzyme

## Abstract

Seed priming is a successful practice to improve crop establishment under adverse environment. However, reduced longevity of primed rice (*Oryza sativa* L.) seeds during storage limited the adoption of this technique. Present study investigated the effect of temperature, relative air humidity (RH) and oxygen on the longevity of primed rice seeds in a range of 60 days storage. In addition, the biochemical and morphological mechanisms associated with deterioration of primed seeds during storage were explored. Three types of priming treated rice seeds and one non-primed control were stored under (1) low temperature-vacuum (LT-V), (2) room temperature-vacuum (RT-V), (3) room temperature-aerobic-low RH (RT-A-LH) and (4) room temperature-aerobic- high RH (RT-A-HH) for 0, 15, 30, 45, and 60 days. The results showed that storage of seeds under different conditions for 15–60 days did not influence the longevity of non-primed rice seeds. Meanwhile, the viability of primed rice seeds did not reduce when stored under LT-V, RT-V, and RT-A-LH, but was significantly reduced under RT-A-HH. Under vacuum condition, the increases of storage temperature (30°C) did not reduce the longevity of primed seeds. Likewise, the oxygen did not influence the longevity of primed rice seeds stored under low RH. Nevertheless, increase of RH significantly reduced the viability of primed seeds stored for 15–60 days. Reduced starch metabolism, the consumption of starch reserves in rice endosperms, the accumulation of malondialdehyde and the decreases of antioxidant enzyme activities might be associated with the deterioration of primed rice seeds during storage. In conclusion, storage of primed seeds under high RH condition beyond 15 days is deteriorative for germination and growth of rice. The primed rice seeds are recommended to store at vacuum or low RH or low temperature condition to ensure good crop establishment.

## Introduction

Seed priming refers to the seed treatment that hydrating the seeds before sowing to activate several metabolic events, but radicle protrusion does not occur (Heydecker and Coolbear, 1977). Several reports have been proved that seed priming was effective in promoting seed germination, enhancing seedling growth of rice (*Oryza sativa* L.) under chilling (Hussain et al., 2016a; Wang et al., 2016), drought (Zheng et al., 2016), and waterlogging stresses (Hussain et al., 2016b). Nevertheless, viability losses of primed seeds during storage is a major limiting factor to wide adoption of seed priming technique.

It has been suggested that the seed longevity is influenced by several factors such as temperature, oxygen, relative air humidity and seed moisture content (Rajjou and Debeaujon, 2008). Our previous research found that the germination percentage and growth attributes of primed rice seedlings were significantly reduced when stored at 25?, while no significant decrease was found when primed seeds were store at −4°C (Hussain et al., 2015). However, the research of Hussain et al. (2015) ignored the influence of oxygen concentration and the relative air humidity (RH) during storage, which are known to greatly impact the seed longevity.

Oxygen has been suggested to be an important factor to regulate series of physiological processes caused by respiration, which was associated with seed deterioration. It has been reported that O_2_ accelerated the accumulation of super oxide and lead to O_2_ injury to seeds during storage (Hendry, 1993). Ellis et al. (2008) reported that oxygen increased the respiration rate and accelerated the seed deterioration at high temperature. Wilson and McDonald (1986) observed that high oxygen concentration accelerated seed deterioration by depleting protective antioxidants. Likewise, RH is also considered as a major factor that control the longevity of the seed in processing and storage (Justice and Bass, 1978). High RH along with high temperature quickly accelerate the deterioration of the seed and thereby cause aging. Therefore, exposure of seed to high temperature and high moisture condition have been wildly used in the controlled deterioration (CD) test by many researchers. Low RH largely reduced the seed deterioration even with high storage temperature (Suma et al., 2013), suggesting that RH might be more important than the storage temperature in determine the seed longevity. Besides, RH has been reported to be interacts with oxygen in determining the seeds longevity during storage. Schwember and Bradford (2011) reported that the presence of O_2_ severely decreased the longevity of primed seeds under low RH, while such phenomena was not observed under high RH. Although the influence of temperature, RH and oxygen concentration on the longevity of normal seeds during storage have been widely studied for several years. The effect of these environmental factors on the longevity of primed rice seeds has rarely been studied.

Starch metabolism is one of the major metabolic event and driving force during rice seed germination. It has been widely reported that the rice seeds germination was significantly correlated with the starch metabolism in rice seeds. Several researches have observed significant reduction of α-amylase activity and total soluble sugar contents in aged seeds (Garcia et al., 2006; Goyoagaa et al., 2011), suggesting that the restriction of starch metabolism might be responsible for the reduced seed germination after storage. For primed seeds, Hussain et al. (2015) observed significant decreases in starch metabolism of primed seeds stored under 25°C, while such decreases were not observed when primed seeds were stored under −4°C. In spite of starch metabolism, the consumption of food reserves in rice endosperm might also be responsible for the viability loss of seeds during storage. Besides, Catusse et al. (2011) reported that in sugar beet, the abundance of alpha-glucosidase was increased by seed priming but down-regulated by seed aging treatment, which reflected that alpha-glucosidase can be used as an indicator for seed priming and aging. However, research concerning the changes of α-amylase activity, total soluble sugar content and the ultrastructure of primed rice seed in response to different storage conditions and storage durations is lacked.

Lipid peroxidation, which is mainly triggered by reactive oxygen species (ROS) in plants cell, could interrupt plants metabolic functions and lead to cell death (Hussain et al., 2016a). Malondialdehyde (MDA) is the end product of lipid peroxidation and could also cause cell damage by reacting with macromolecules (Xu et al., 2015). On the other side, the antioxidant defense systems, which include the antioxidant enzymes such as peroxidase (POD), superoxide dismutase (SOD), and catalase (CAT), have been regarded as the most important defense system to protect the plants from oxidative stress by eliminating the accumulation of ROS (Foyer and Noctor, 2005; Gill and Tuteja, 2010). The strong correlation between antioxidant system and seed aging has been reported by several researches (Zhu and Chen, 2007; Demirkaya et al., 2010). Sung and Jeng (2010) suggested that the ROS could be accumulated during storage with the anticipation of oxygen and water. Under mild aging condition (natural aging), the abundance of CAT enzyme in Arabidopsis seeds was largely decreased (Rajjou et al., 2008). For primed seeds, the changes of CAT abundance in response to seed priming and aging have been reported in sugar beet (Catusse et al., 2011). However, the changes of lipid peroxidation level and antioxidant activity in primed rice seeds under various natural storage conditions and durations is still lacking.

Understanding the influences of different storage conditions on longevity of primed rice seeds could provide valuable information on the application of seed priming technology. The aims of this study were (1) to evaluate the effects of temperature (low temperature and room temperature), oxygen (aerobic and vacuum) and RH (low RH and high RH) on germination and seedling growth attributes of primed and non-primed rice seeds; (2) to explore the biochemical and morphological changes of primed and non-primed rice seeds stored under different storage conditions and durations.

## Materials and methods

### Seed source

The rice (*O. sativa* L.) seeds of a local mega variety Huanghuazhan (HHZ, inbred) were used in present experiment. The initial germination percentage of the seeds was over 95%.

### Seed priming treatment

The process of seed priming is soaking the dry seeds into the solution which contained priming agent for 24 h and then air-dried until the seed moisture content reduced to <10%. The seed priming treatments selected for this study were hydro-priming (distilled water, HP), osmopriming (10% polyethylene glycol, PEG) and spermidine priming (0.5 mmol L^−1^, Spd). Seeds were primed in the dark at 25°C for 24 h, with constant gentle agitation, the ratio of seed weight to solution volume (w/v) was 1:5. The priming solution was changed after every 12 h (Zheng et al., 2016). The non-primed seeds were used as control. After 24 h, the primed seeds were washed with distilled water for 2 min, surface dried by blotting paper and transferred to air dry oven at 25? for 48 h to reduce the moisture content (Hussain et al., 2015). Dried primed and non-primed seeds were then subjected to different seed storage conditions and durations.

### Storage treatment

Primed and non-primed seeds were stored at four different storage conditions: (1) Low temperature-vacuum (LT-V); (2) Room temperature-vacuum (RT-V); (3) Room temperature-aerobic-low RH (RT-A-LH); (4) Room temperature-aerobic-high RH (RT-A-HH). For vacuum treatment, the primed and non-primed rice seeds seed were packed and sealed in aluminum foil bags coated with polyethylene using vacuum sealer. After that, the vacuumed seed bags were transferred to a freezer (−4?, LT-V) or stored at room temperature (RT-V). For RT-A-LH treatment, the primed and non-primed rice seeds were packed in mesh bags and stored in glass desiccator (the RH is around 25%). For RT-A-HH treatment, the primed and non-primed rice seeds were packed in mesh bags and stored at room temperature (30°C). The primed and non-primed seeds were immediately stored under different storage conditions for 0, 15, 30, 45, 60 days, respectively. The average temperatures and RH of each storage condition and duration were presented in Table 1.

**Table 1:** Mean temperature, mean relative humidity, and oxygen availability of four storage conditions and four storage durations.

**Storage condition**	**Storage duration (days)**	**Temperature (°C)**	**Relative humidity (%)**	**Aerobic or vacuum**
LT-V	1	−4.00	0	Vacuum
	30			
	45			
	60			
RT-V	15	29.79	0	Vacuum
	30	30.64		
	45	31.12		
	60	30.81		
RT-A-LH	15	29.79	26.00	Aerobic
	30	30.64	24.56	
	45	31.12	23.73	
	60	30.81	22.81	
RT-A-HH	15	29.79	56.96	Aerobic
	30	30.64	62.87	
	45	31.12	64.62	
	60	30.81	66.68	

*LT-V, the primed and non-primed seeds were stored under vacuum, low temperature condition; RT-V, the primed and non-primed seeds were stored under vacuum, room temperature condition; RT-A-LH, the primed and non-primed seeds were stored under room temperature, aerobic and low relative humidity condition; RT-A-HH, the primed and non-primed seeds were stored under room temperature, aerobic and high relative humidity condition*.

### Experimentation

The experiments were conducted during 2016. Forty healthy seeds of each treatment were evenly placed on two layers of filter paper in petri dishes (14.5 cm diameter). After adding 20 ml water to each treatment, The petri dishes were transferred to growth chamber with 12 h light period, and 30°C (day): 25°C (night temperature). Equal volume of distilled water was applied to all petri dishes when their moisture content decreased. All the experiments were laid out in a completely randomized design with four biological replications.

### Observation

The rice seeds germination was recorded based on the instruction of Association of Official Seed Analysis (1990). Germination percentage was calculated as the ratio of the number of seeds germinated to the total number of seeds. Germination index (GI) was calculated as GI = $\frac{No.\;of\;emerged\;seeds}{Days\;of\;first\;count}+$———$+\frac{No.\;of\;emerged\;seeds}{Days\;of\;final\;count}$. Vigor index (VI) was calculated by multiplying GI with seedling length. At 6 days after sowing (DAS), 10 seedlings were randomly sampled from each treatment and each replication. The root length and fresh weight as well as shoot length and fresh weight of each sample was recorded immediately. Each observation was replicated for four times.

For the determination of α-amylase activity, 0.3 g frozen seedlings (each treatment was replicated for four times) were homogenized and rinsed with 8 ml ice-cold Na-phosphate buffer (pH 7.0, 0.1 M). After centrifuging at 12,000 g for 20 min, the supernatant was collected as a crude extract. The DNS method was used to determine the α-amylase activity (Bernfeld, 1955).

The determination of the total soluble sugar contents was according to the methods of Zheng et al. (2016), 0.3 g frozen seedling samples of each treatment (with four biological replications) were ground and mixed with 50 ml distilled water. Then the mixture was filtered using Whatman No. 42 filter paper. The total soluble sugar contents in rice seedlings were evaluated by the phenol sulfuric method (Dubois et al., 1956).

The scanning electron microcopy assay was conducted to examine the morphology changes of primed rice seeds after storage. As three priming treatments showed similar response to different storage condition and storage duration. Only one priming treatment (hydro-priming, HP) was selected to conduct the scanning electron microcopy assay. The primed rice seeds that stored under four different conditions for 0, 30 and 60 days were incubated at 25? for 12 h and then soaked in liquid nitrogen for 2 min (Pallwall et al., 1991). The endosperm of the primed rice seeds was cut longitudinally in half with a razor blade and then dehydrated with a series of 30, 50, 70, 80, 90, 95, 100% ethanol and dried to critical point in a critical-point drying apparatus. After then, the dehydrated samples were coated with gold- palladium for 20 min in a coating machine. The structure changes of rice endosperms were observed in a scanning electron microscope.

For the lipid peroxidation and antioxidant enzymes assay, rice seeds were incubated at 25? for 24 h, then 0.5 g fresh samples in each container were grounded with 8 ml of 50 mM phosphate buffer (pH 7.0, containing 1% (w/v) polyvinylpyr rolidone) (Zheng et al., 2016). After centrifuging at 15,000 g for 20 min at 4?. The supernatant was collected as crude extracts for the determination of the SOD, POD, CAT activity and the MDA content. Each assay was replicated for four times. The malondialdehyde (MDA) content was determined by using the method of Zheng et al. (2016). Two milliliter enzyme crude extracts was mixed with 1 ml reaction liquids (consisted of 20% trichloroacetic acid and 0.5% thiobarbituric acid). Then the mixture was incubated at 95? for 20 min. After cooling to room temperature, the mixture was centrifuged at 10,000 g for 10 min. The absorbance of supernatant at 532 nm was determined and the nonspecific absorbance at 600 nm was subtracted. The MDA content was calculated by the extinction coefficient of 155 mM^−1^ cm^−1^ (Zheng et al., 2016).

The CAT activity was determined by the method of Zheng et al. (2016) and Beers and Sizer (1952). 0.5 ml enzyme crude extract was mixed with of 2 ml of sodium phosphate buffer (50 mM, pH 7.0), and 0.5 ml hydrogen peroxide (40 mM). Then the decline in absorbance at 240 nm was immediately recorded using ultraviolet spectrophotometer (Tecan infinite M200, Switzerland) (Beers and Sizer, 1952). The CAT activity was defined as an absorbance change of 0.01 units per min and expressed as U g^−1^ FW (Zheng et al., 2016).

The POD activity was determined by guaiacol oxidation method with slight modifications (Chance and Maehly, 1995; Zheng et al., 2016). 0.1 ml crude extract was reacted with 2.9 ml reaction mixture which contains 50 mM sodium acetate buffer (pH 5.0), 20 mM guaiacol and 40 mM hydrogen peroxide, and in the blank control, the enzyme extract was replaced by sodium acetate buffer. The absorbance changes at 470 nm were recorded using ultraviolet spectrophotometer (Tecan infinite M200, Switzerland) within 3 min after the start of the reaction. One unit POD activity was defined as an absorbance change of 0.01 units per min and was expressed as U g^−1^ FW (Zheng et al., 2016).

The SOD activity was evaluated by nitro blue tetrazolium (NBT) methods with slight modifications (Zheng et al., 2016). 0.3 ml enzyme crude extract was reacted with 2.7 ml reaction mixture that consisted of 1.5 ml phosphate buffer (50 mM, pH 7.8), 0.3 ml methionine (130 mM), 0.3 ml NBT (750 μM), 0.3 ml EDTA [100 μM), 0.3 ml riboflavin (20 μM)] (Zheng et al., 2016). The reaction solution were irradiated under a light bar (15 fluorescent lamps) at 78 μmol m^−2^ s^−1^ for 15 min. The absorbance of the irradiated and non-irradiated solution at 560 nm was determined with a spectrophotometer (Tecan infinite M200, Switzerland). One unit of SOD activity was defined as the amount of enzyme that gives 50% inhibition of NBT photoreduction and was presented as U g^−1^ FW (Zheng et al., 2016).

### Statistics analysis

All data from present experiment are expressed as the mean value of four biological replications. Statistix 11.0 was used to analyze the data using least significant difference (LSD) test at 0.05 probability level.

## Results

### Germination

**Figure 1:**
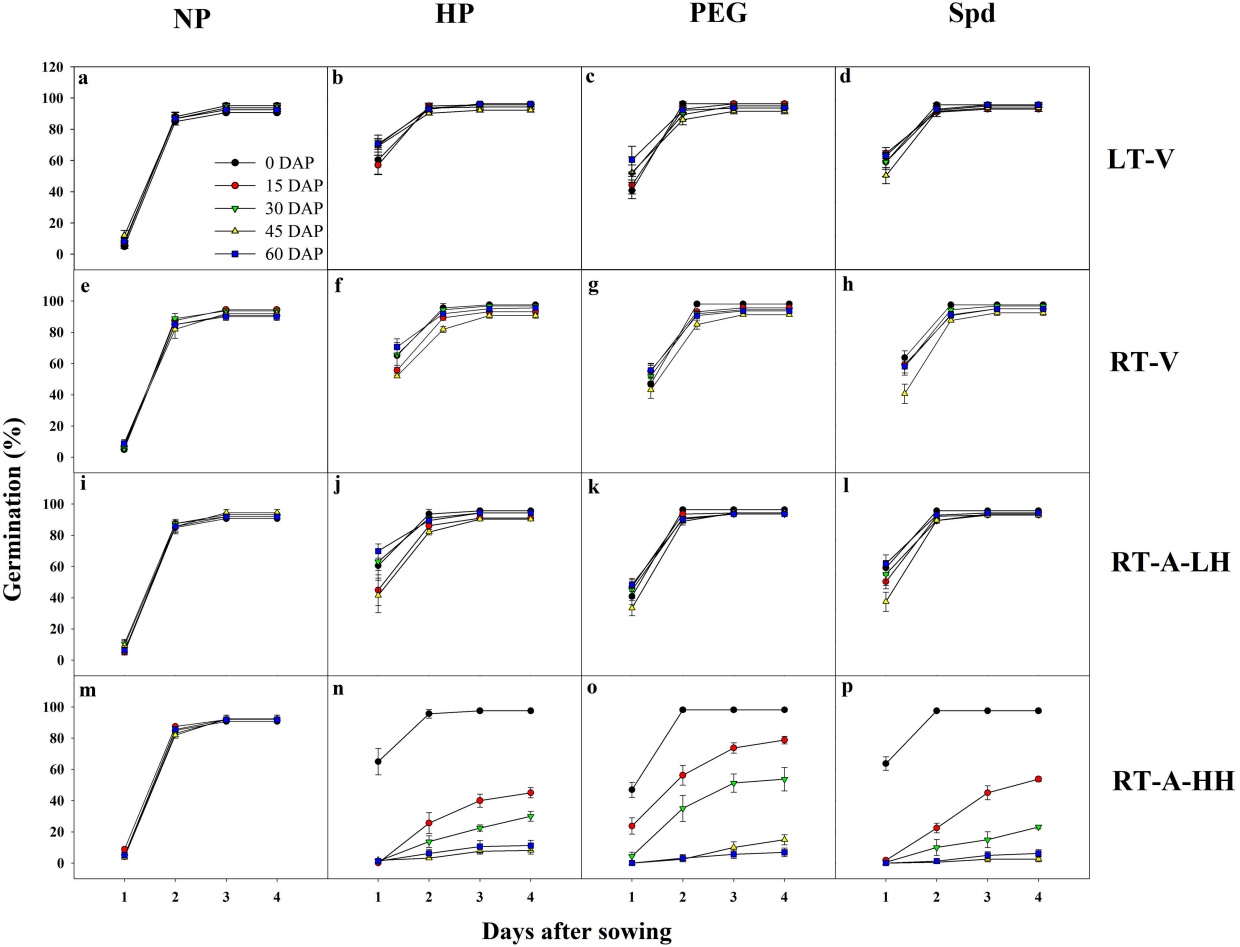
Germination dynamics of primed and non-primed rice seeds stored under different storage conditions and storage durations. **(a–d)** Seeds of NP, HP, PEG and Spd stored under LT-V, respectively. **(e–h)** Seeds of NP, HP, PEG, and Spd stored under RT-V, respectively. **(i–l)** Seeds of NP, HP, PEG, and Spd stored under RT-A-LH, respectively. **(m–p)** Seeds of NP, HP, PEG, and Spd stored under RT-A-HH, respectively. DAP, days after priming; NP, non-primed control; HP, hydro-priming; PEG, osmopriming; Spd, spermidine priming; LT-V, the primed and non-primed seeds were stored under vacuum; low temperature condition; RT-V, the primed and non-primed seeds were stored under vacuum, room temperature condition; RT-A-LH, the primed and non-primed seeds were stored under room temperature, aerobic and low relative humidity condition; RT-A-HH, the primed and non-primed seeds were stored under room temperature, aerobic and high relative humidity condition. Error bar above mean indicate stand error (*n* = 4).

Germination dynamics of primed and non-primed rice seeds regarding different storage conditions and storage durations were presented in Figure 1. For non-primed seeds, no significant difference was found on germination dynamics among various storage conditions and storage durations. Similarly, the germination of the primed seeds stored under LT-V, RT-V and RT-A-LH did not decrease after 60 days of storage. However, under RT-A-HH, significant decreases in germination speed and germination percentage was observed after 15 days of storage, and the germination decreased drastically with prolonged storage durations. All three priming treatments showed similar responses to different storage conditions and durations.

Significant (*P* < 0.05) variations of storage condition and storage duration on germination attributes of primed rice seeds were recorded (Table 2). When averaged across three priming treatments, the germination percentage, GI and VI of primed seeds stored under LT-V for 60 days were 97.1, 69.2, and 261.3, respectively, which showed no significant difference to that of the un-stored primed seeds. Likewise, the germination attributes of the primed seeds stored under RT-V and RT-A-LH were not decreased during 0–60 days of storage. However, when the primed-seeds were stored under RT-A-HH, all the germination attributes were considerably decreased. When averaged across three priming treatments, the germination percentage, GI and VI of the primed seeds stored under RT-A-HH for 15 days were decreased by 39.4, 64.4, and 83.3% respectively as compared with that of un-stored primed seeds. When the storage duration extended to 60 days, the germination percentage, GI and VI of primed seeds stored under RT-A-HH were decreased by 88.9, 95.3, and 99.6%, respectively as compared with un-stored primed seeds.

**Table 2:** Germination percentage, germination index, and vigor index of primed and non-primed seeds stored under different storage conditions and storage durations.

**Priming treatment**	**Storage condition**	**Storage duration (days)**	**Germination (%)**	**Germination index**	**Vigor index**
NP	LT-V	0	90.6b	40.15b	110.12ab
		15	95.0a	42.58a	118.91a
		30	95.0a	42.83a	117.63a
		45	93.8ab	44.03a	113.95ab
		60	92.5ab	42.23ab	100.20b
		Mean	93.4A	42.36A	112.11AB
	RT-V	0	90.6a	40.15a	110.05a
		15	94.4a	42.31a	111.53a
		30	93.8a	41.92a	124.55a
		45	91.9a	41.08a	119.78a
		60	90.0a	41.53a	117.60a
		Mean	92.1AB	41.39AB	116.70A
	RT-A-LH	0	90.6a	40.15a	110.03a
		15	93.1a	41.23a	113.78a
		30	91.9a	43.19a	109.78a
		45	94.4a	42.90a	114.36a
		60	91.9a	41.06a	105.67a
		Mean	92.4AB	41.72A	110.73AB
	RT-A-HH	0	90.6a	40.15a	110.03a
		15	91.9a	40.58ab	110.05ab
		30	91.9a	40.15ab	100.55ab
		45	91.9a	39.83ab	98.35ab
		60	90.6a	39.48b	93.97b
		Mean	91.8B	40.50B	103.45B
HP	LT-V	0	97.5ab	67.90a	277.20a
		15	97.5ab	66.90a	250.77a
		30	96.3ab	71.10a	277.05a
		45	94.4b	69.82a	266.75a
		60	98.1a	71.68a	281.45a
		Mean	96.8A	69.48A	270.64A
	RT-V	0	97.5a	67.93a	277.20a
		15	93.1ab	61.97ab	222.42bc
		30	96.9a	67.80a	251.15ab
		45	90.6b	58.28b	212.18c
		60	95.6a	68.91a	270.62a
		Mean	94.8B	64.94B	246.71A
	RT-A-LH	0	97.5a	67.93a	277.20a
		15	93.1b	59.75b	236.38bc
		30	96.3a	68.13a	279.55a
		45	92.5b	57.62b	217.58c
		60	96.3a	70.38a	274ab
		Mean	95.1AB	64.75B	256.94A
	RT-A-HH	0	97.5a	67.92a	277.20a
		15	45.0b	14.98b	24.90b
		30	30.0c	11.78b	10.61bc
		45	8.1d	3.23c	3.40bc
		60	11.3d	4.31c	0.63c
		Mean	38.5C	20.44C	63.34B
PEG	LT-V	0	98.1a	61.28a	220.83a
		15	98.1a	61.92a	228.55a
		30	96.9ab	63.75a	239.37a
		45	93.8b	60.15a	212.93a
		60	95.6ab	67.23a	243.08a
		Mean	96.5A	62.86A	228.95A
	RT-V	0	98.1a	61.28a	220.83b
		15	95.6ab	62.98a	242.72ab
		30	94.4b	61.18a	226.95ab
		45	91.3c	55.55b	191.53c
		60	93.8bc	62.28a	243.22a
		Mean	94.6A	60.65B	225.05A
	RT-A-LH	0	98.1a	61.28ab	220.83a
		15	96.3a	62.63a	229.70a
		30	95.6a	61.23ab	228.13a
		45	96.3a	56.73b	221.75a
		60	95.6a	62.35a	220.07a
		Mean	96.4A	60.84B	224.09A
	RT-A-HH	0	98.1a	61.28a	220.83a
		15	78.8b	38.50b	78.13b
		30	53.8c	20.95c	27.22c
		45	6.9d	2.08d	1.85d
		60	15.0d	3.35d	2.15d
		Mean	50.5B	25.23C	66.03B
Spd	LT-V	0	97.5a	67.78a	266.38a
		15	95.0a	68.33a	248.13a
		30	96.9a	67.03a	246.40a
		45	95.6a	63.33a	232.97a
		60	97.5a	68.75a	259.50a
		Mean	96.5A	67.04A	250.68A
	RT-V	0	97.5a	67.78a	266.38a
		15	95.0ab	64.08a	240.65a
		30	96.9ab	64.75a	238.22a
		45	92.5b	55.35b	185.50b
		60	95.0ab	63.68a	227.08ab
		Mean	95.4A	63.13B	231.56B
	RT-A-LH	0	97.5a	67.78a	266.38a
		15	95.0a	63.33ab	232.82bc
		30	95.6a	64.70a	242.65ab
		45	95.0a	58.08b	207.10c
		60	96.3a	67.98a	260.43a
		Mean	95.8A	64.37B	241.87AB
	RT-A-HH	0	97.5a	67.78a	266.38a
		15	53.8b	16.65b	24.75b
		30	23.1c	6.58c	6.60bc
		45	2.5d	0.73d	0.00c
		60	6.3d	1.53d	0.00c
		Mean	36.6B	18.65C	59.55C
	*P*-values	Priming treatment	*P* < 0.01	*P* < 0.01	*P* < 0.01
		Storage condition	*P* < 0.01	*P* < 0.01	*P* < 0.01
		Storage duration	*P* < 0.01	*P* < 0.01	*P* < 0.01

*Different lowercase letters denote statistical difference between storage durations of a storage condition at the 5% level according to LSD test. Different uppercase letters denote statistical difference between storage conditions at the 5% level according to LSD test. NP, non-primed control; HP, hydro-priming; PEG, osmopriming; Spd, spermidine priming; LT-V, the primed and non-primed seeds were stored under vacuum, low temperature condition; RT-V, the primed and non-primed seeds were stored under vacuum, room temperature condition; RT-A-LH, the primed and non-primed seeds were stored under room temperature, aerobic and low relative humidity condition; RT-A-HH, the primed and non-primed seeds were stored under room temperature, aerobic and high relative humidity condition*.

### Seedling growth

**Figure 2:**
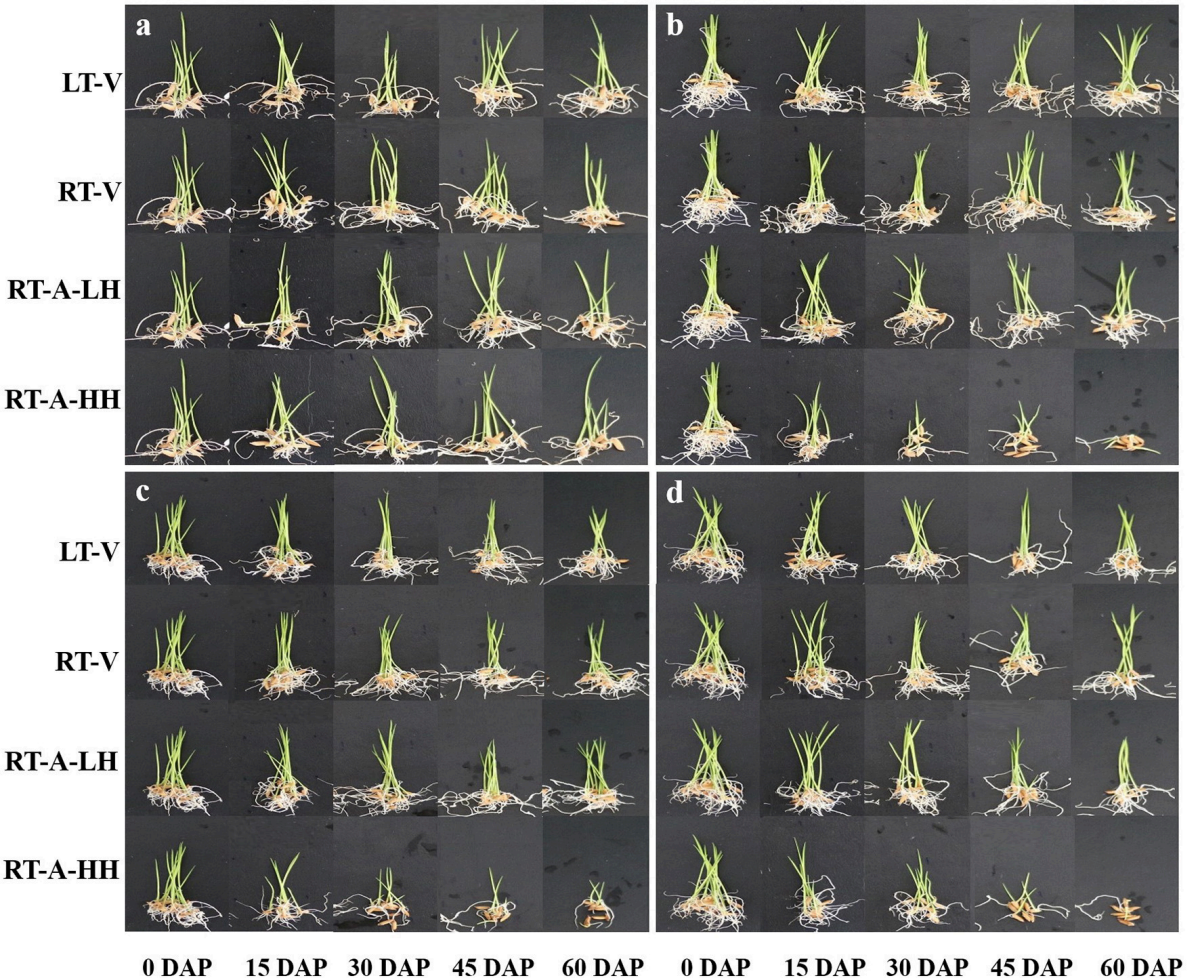
Early growth performance of non-primed and primed rice seedlings under different storage conditions and durations. **(a)** Non-primed control, **(b)** Hydro-priming, **(c)** Osmopriming, **(d)** Spermidine priming. DAP, days after priming; NP, non-primed control; HP, hydro-priming; PEG, Osmopriming; Spd, spermidine priming; LT-V, the primed and non-primed seeds were stored under vacuum, low temperature condition; RT-V, the primed and non-primed seeds were stored under vacuum, room temperature condition; RT-A-LH, the primed and non-primed seeds were stored under room temperature, aerobic and low relative humidity condition; RT-A-HH, the primed and non-primed seeds were stored under room temperature, aerobic and high relative humidity condition. The figure was merged from 52 pictures. All pictures shown were taken under exactly the same conditions.

A pictorial illustration of primed and non-primed rice seedlings (6 DAS) stored under different storage durations and storage conditions is presented in Figure 2. Consistent with germination attributes, no significant difference was found in seedling growth attributes of non-primed rice seedlings stored under different storage conditions and durations (Table 3). For primed rice seeds, significant (*P* < 0.05) effects of storage conditions and storage durations on seedling growth were observed (Table 3). When stored under LT-V, RT-V, and RT-A-LH, the seedling growth attributes did not decrease when seeds were stored for 60 days. Nevertheless, the seedling growth attributes of primed rice seeds stored under RT-A-HH were significantly decreased were stored for 15 days. When averaged across three priming treatments, the root length, root fresh weight, shoot length, shoot fresh weight of the primed rice seedlings stored under RT-A-HH for 15 days were decreased by 58.3, 37.5, 156.7, and 128.7%, respectively. Further increase of storage duration brought concomitant decreases in seedling growth attributes of primed seed. Averaged across three priming treatments, root length, root fresh weight, shoot length and shoot fresh weight of the rice seedlings stored under RT-A-HH were decreased by 69.4–95.8%, 39.3–64.9%, 119.7–230.7%, and 128.7–248.9%, respectively when seed storage duration was prolonged from 15 days to 60 days, compared with that of un-stored control (Table 3).

**Table 3:** Root length, shoot length, root fresh weight, and shoot fresh weight of primed and non-primed seedlings (6 DAS) stored under different storage conditions and storage durations.

**Priming treatment**	**Storage condition**	**Storage duration**	**Root length (cm)**	**Shoot length (cm)**	**Fresh root weight (mg)**	**Fresh shoot weight (mg)**
NP	LT-V	0	4.4a	2.7a	8.34a	11.74a
		15	4.2a	2.8a	9.28a	11.20a
		30	4.4a	2.7a	9.84a	12.22a
		45	4.0a	2.6a	8.63a	11.14a
		60	4.7a	2.4a	8.08a	10.37a
		Mean	4.4A	2.6A	8.82A	11.33A
	RT-V	0	4.4a	2.7a	8.34a	11.74a
		15	4.5a	2.6a	9.05a	11.08a
		30	4.4a	3.0a	9.31a	12.26a
		45	4.0a	2.9a	8.15a	11.81a
		60	3.6a	2.8a	7.68a	11.84a
		Mean	4.2AB	2.8A	8.51AB	11.75A
	RT-A-LH	0	4.4a	2.7a	8.34a	11.74a
		15	3.8a	2.8a	9.14a	10.70a
		30	4.5a	2.5a	8.15a	11.65a
		45	4.5a	2.6a	6.47a	10.97a
		60	3.8a	2.6a	7.25a	11.20a
		Mean	4.2AB	2.7A	7.93B	11.25A
	RT-A-HH	0	4.4a	2.7a	8.34a	11.74a
		15	3.8ab	2.7a	7.81a	11.67a
		30	3.6b	2.4a	8.07a	11.60a
		45	4.3ab	2.5a	9.16a	11.15a
		60	4.3ab	2.5a	7.74a	10.87a
		Mean	4.1B	2.6A	8.22AB	11.41A
HP	LT-V	0	6.0a	4.1a	13.66a	16.07a
		15	5.5a	3.8a	11.73a	14.02a
		30	5.2a	3.9a	13.08a	15.45a
		45	5.9a	3.8a	13.68a	14.03a
		60	6.2a	3.9a	14.21a	16.30a
		Mean	5.8A	3.9A	13.28A	15.19A
	RT-V	0	6.0ab	4.1a	13.66ab	16.07a
		15	4.9b	3.6a	11.78b	14.25a
		30	5.4ab	3.7a	13.91ab	14.20a
		45	5.0ab	3.6a	11.79b	13.69a
		60	6.0a	3.9a	14.79a	15.38a
		Mean	5.5A	3.8A	13.20A	14.73A
	RT-A-LH	0	6.0a	4.1a	13.66ab	16.07a
		15	5.4a	3.9a	13.79ab	14.15a
		30	5.6a	4.1a	14.00a	15.00a
		45	5.4a	3.8a	13.08b	13.99a
		60	5.5a	3.9a	13.71ab	14.77a
		Mean	5.6A	4.0A	13.67A	14.80A
	RT-A-HH	0	6.0a	4.1a	13.66a	16.07a
		15	1.8b	1.7b	2.80b	7.04b
		30	1.4c	1.0c	2.41bc	6.14c
		45	1.1c	1.1c	0.91c	1.96c
		60	0.2c	0.1c	0.45c	0.89c
		Mean	2.1B	1.6B	4.05B	6.44B
PEG	LT-V	0	5.5a	3.6a	12.72a	15.22a
		15	5.7a	3.7a	12.98a	15.13a
		30	5.5a	3.8a	13.30a	14.04a
		45	5.4a	3.6a	14.55a	14.89a
		60	5.8a	3.6a	13.08a	13.99a
		Mean	5.6A	3.6A	13.34A	14.66A
	RT-V	0	5.5a	3.6a	12.72c	15.22a
		15	5.7a	3.9a	14.14a	15.54a
		30	5.9a	3.7a	13.43ab	14.48a
		45	5.4a	3.5a	13.02bc	14.05a
		60	5.8a	3.9a	13.22abc	14.42a
		Mean	5.6A	3.7A	13.31A	14.75A
	RT-A-LH	0	5.5ab	3.6a	12.72a	15.22a
		15	5.8ab	3.7a	13.10a	14.31a
		30	5.4ab	3.7a	14.84a	14.78a
		45	5.2b	3.9a	14.04a	14.83a
		60	5.9a	3.5a	13.18a	13.69a
		Mean	5.6A	3.7A	13.58A	14.57A
	RT-A-HH	0	5.5a	3.6a	12.72a	15.22a
		15	3.1b	2.1b	7.57b	9.82b
		30	1.5c	1.2c	3.52bc	5.45c
		45	0.5c	0.4c	1.47c	2.23c
		60	0.3c	0.6c	0.49c	1.93c
		Mean	2.2B	1.6B	5.16B	6.94B
Spd	LT-V	0	5.2ab	3.9a	13.32a	13.35a
		15	4.9b	3.6a	12.30a	12.93a
		30	4.9b	3.7a	13.48a	14.28a
		45	5.2ab	3.7a	13.40a	13.82a
		60	5.6a	3.8a	12.72a	13.82a
		Mean	5.1A	3.7A	13.05A	13.65A
	RT-V	0	5.2a	3.9a	13.32a	13.35a
		15	5.1a	3.8a	13.27a	14.06a
		30	4.8ab	3.7a	12.27a	13.16a
		45	4.3b	3.3a	12.03a	12.75a
		60	5.3a	3.6a	12.52a	13.56a
		Mean	5.0A	3.8A	13.70A	13.40A
	RT-A-LH	0	5.2ab	3.9a	13.32a	13.35a
		15	5.3ab	3.7a	13.31a	14.82a
		30	5.1ab	3.8a	13.08a	13.92a
		45	4.6b	3.6a	12.28a	13.70a
		60	5.9a	3.8a	13.66a	14.63a
		Mean	5.2A	3.8A	13.13A	14.10A
	RT-A-HH	0	5.2a	3.9a	13.32a	13.35a
		15	2.0b	1.5b	3.01b	6.16b
		30	0.7c	0.8b	2.22c	2.94c
		45	0.0d	0.0c	0.00d	0.00d
		60	0.1d	0.0c	0.00d	0.00d
		Mean	1.6B	1.3B	3.72B	4.50B

*Different lowercase letters denote statistical difference between storage durations of a storage condition at the 5% level according to LSD test. Different uppercase letters denote statistical difference between storage conditions at the 5% level according to LSD test. DAS, days after sowing; NP, non-primed control; HP, hydro-priming; PEG, osmopriming; Spd, spermidine priming; LT-V, the primed and non-primed seeds were stored under vacuum, low temperature condition; RT-V, the primed and non-primed seeds were stored under vacuum, room temperature condition; RT-A-LH, the primed and non-primed seeds were stored under room temperature, aerobic and low relative humidity condition; RT-A-HH, the primed and non-primed seeds were stored under room temperature, aerobic and high relative humidity condition*.

### Starch metabolism

**Figure 3:**
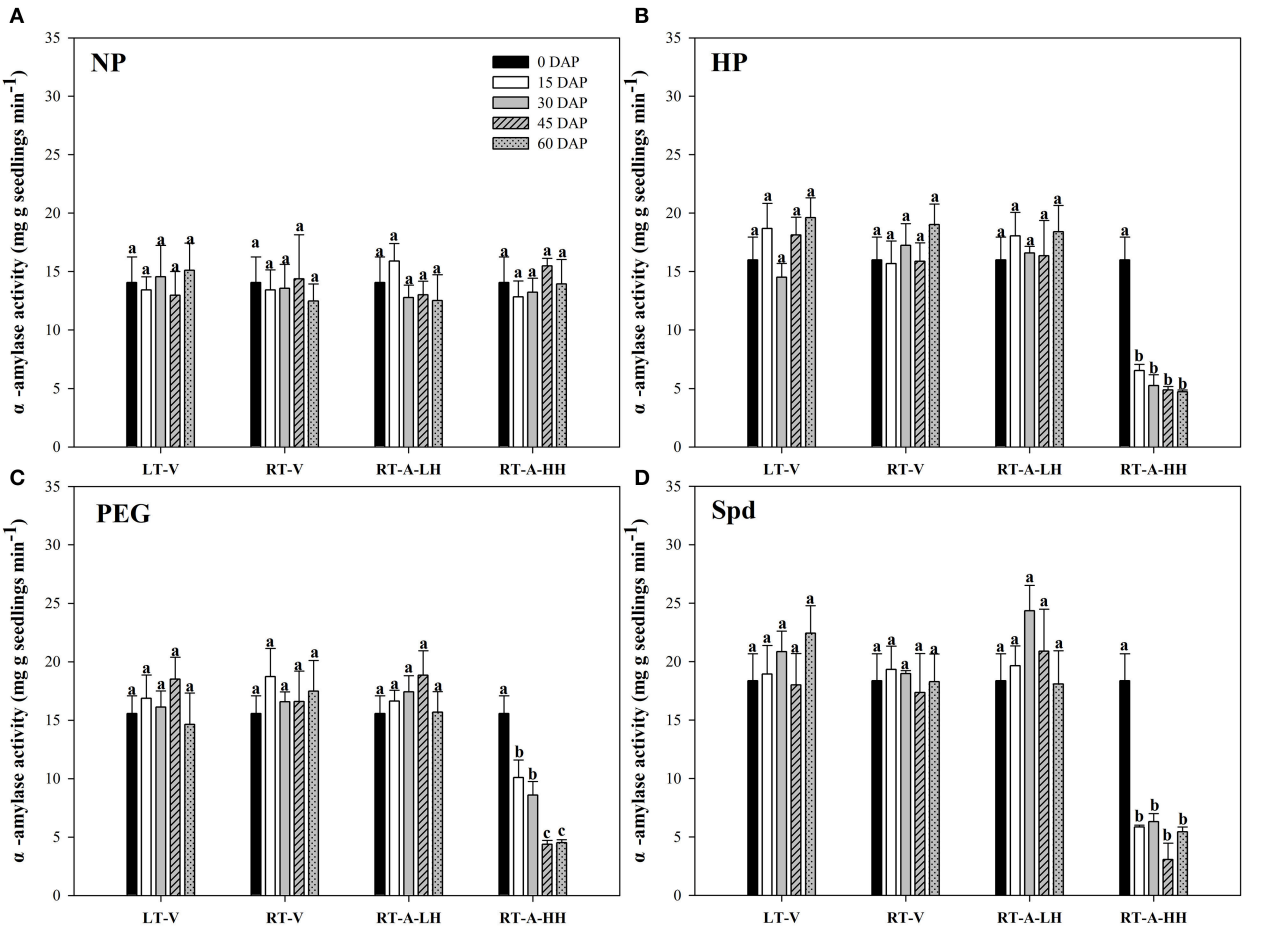
Variations in α-amylase activity of rice seeds and seedlings in primed and non-primed rice seedlings (6 DAS) under different storage conditions and storage durations. **(A)** Non-primed control, **(B)** Hydro-priming, **(C)** Osmopriming, **(D)** Spermidine priming. DAS, days after sowing; DAP, days after priming; NP, non-primed control; HP, hydro-priming; PEG, Osmopriming; Spd, spermidine priming; LT-V, the primed and non-primed seeds were stored under vacuum, low temperature condition; RT-V, the primed and non-primed seeds were stored under vacuum, room temperature condition; RT-A-LH, the primed and non-primed seeds were stored under room temperature, aerobic and low relative humidity condition; RT-A-HH, the primed and non-primed seeds were stored under room temperature, aerobic and high relative humidity condition. Error bar above mean indicate stand error (*n* = 4). Different letters denote difference between storage durations of a storage condition at the 5% level according to LSD test.

**Figure 4:**
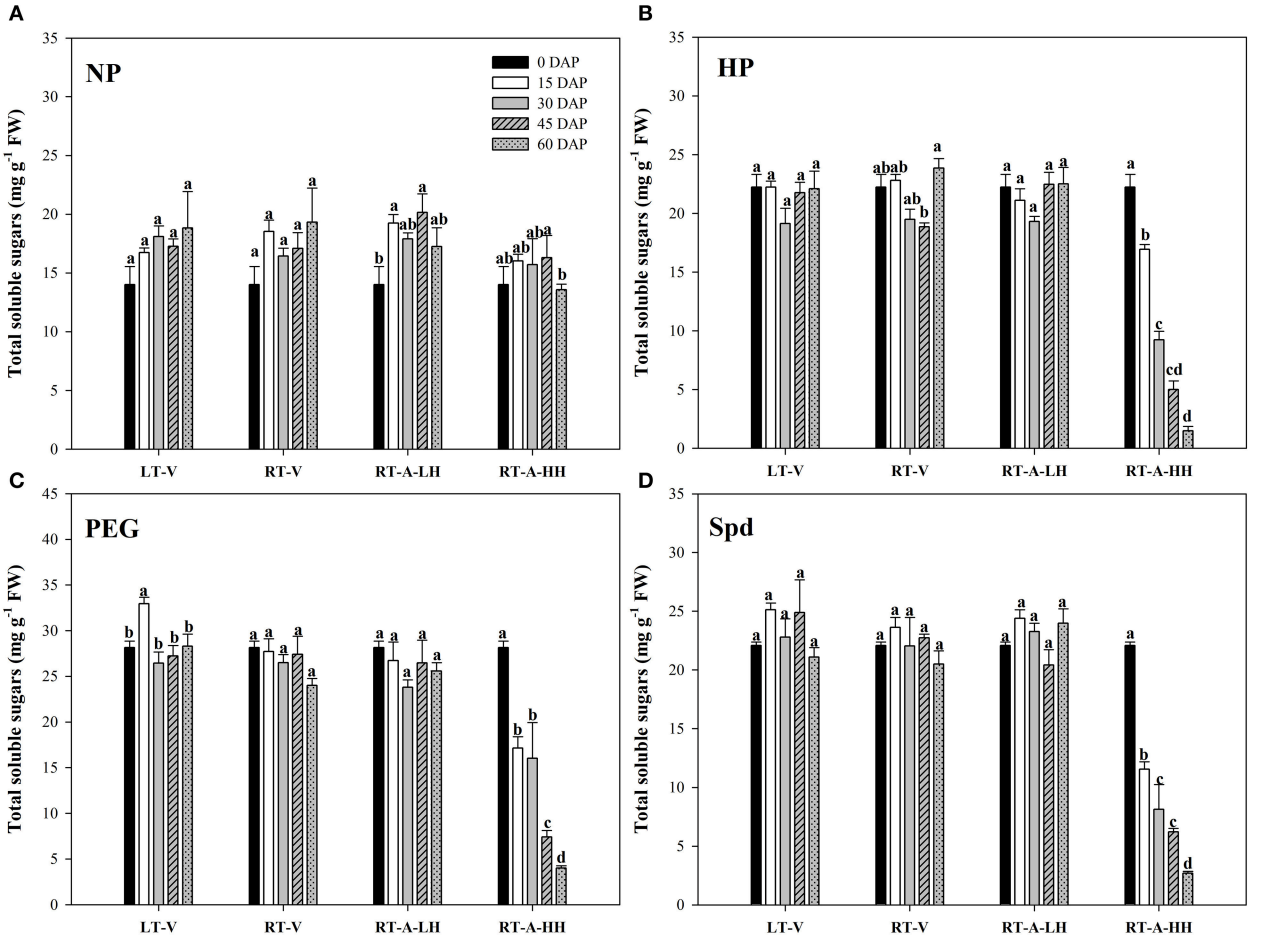
Variations in total soluble sugar content of rice seeds and seedlings in primed and non-primed rice seedlings (6 DAS) under different storage conditions and storage durations. **(A)** Non-primed control, **(B)** Hydro-priming, **(C)** Osmopriming, **(D)** Spermidine priming. DAS, days after sowing; DAP, days after priming; NP, non-primed control; HP, hydro-priming; PEG, Osmopriming; Spd, spermidine priming; LT-V, the primed and non-primed seeds were stored under vacuum, low temperature condition; RT-V, the primed and non-primed seeds were stored under vacuum, room temperature condition; RT-A-LH, the primed and non-primed seeds were stored under room temperature, aerobic and low relative humidity condition; RT-A-HH, the primed and non-primed seeds were stored under room temperature, aerobic and high relative humidity condition. Error bar above mean indicate stand error (*n* = 4). Different letters denote difference between storage durations of a storage condition at the 5% level according to LSD test.

Starch metabolism in rice seeds and seedlings was reflected by the α-amylase activity and total soluble sugar content during seed germination (Figures 3, 4). For the non-primed seeds, α-amylase activity and total soluble sugar content in primed rice seedlings showed no significant difference among different storage durations and storage conditions. In contrary, significant variations (*P* < 0.05) in starch metabolism of primed rice seeds stored under various storage conditions and durations were found. When the primed seeds were stored under LT-V, RT-V, RT-A-LH for 60 days, no significant decreases were observed in α-amylase activity and total soluble sugar content in rice seedlings, as compared with un-stored control. However, α-amylase activity and total soluble sugar content in rice seedlings were significantly decreased when stored at RT-A-HH for even only 15 days. When averaged across priming treatments, the α-amylase activity in rice seedlings decreased by 55.0, 59.6, 75.3, and 70.6% stored for 15–60 days respectively, as compared with 0 DAP control. The total soluble sugar contents in primed rice seedlings showed the similar trends to that of α-amylase activity. The seeds stored under RT-A-HH exhibited the lowest α-amylase activity and total soluble sugar content among four storage conditions. When averaging across priming treatments and storage durations, the α-amylase activity of rice seedlings stored under RT-A-HH was decreased by 55.1, 54.0, and 55.7%, respectively, while the total soluble sugar content was decreased by 51.3, 49.3, and 49.4% respectively as compared with that under LT-V, RT-V, RT-A-LH.

### Scanning electron microscopy

**Figure 5:**
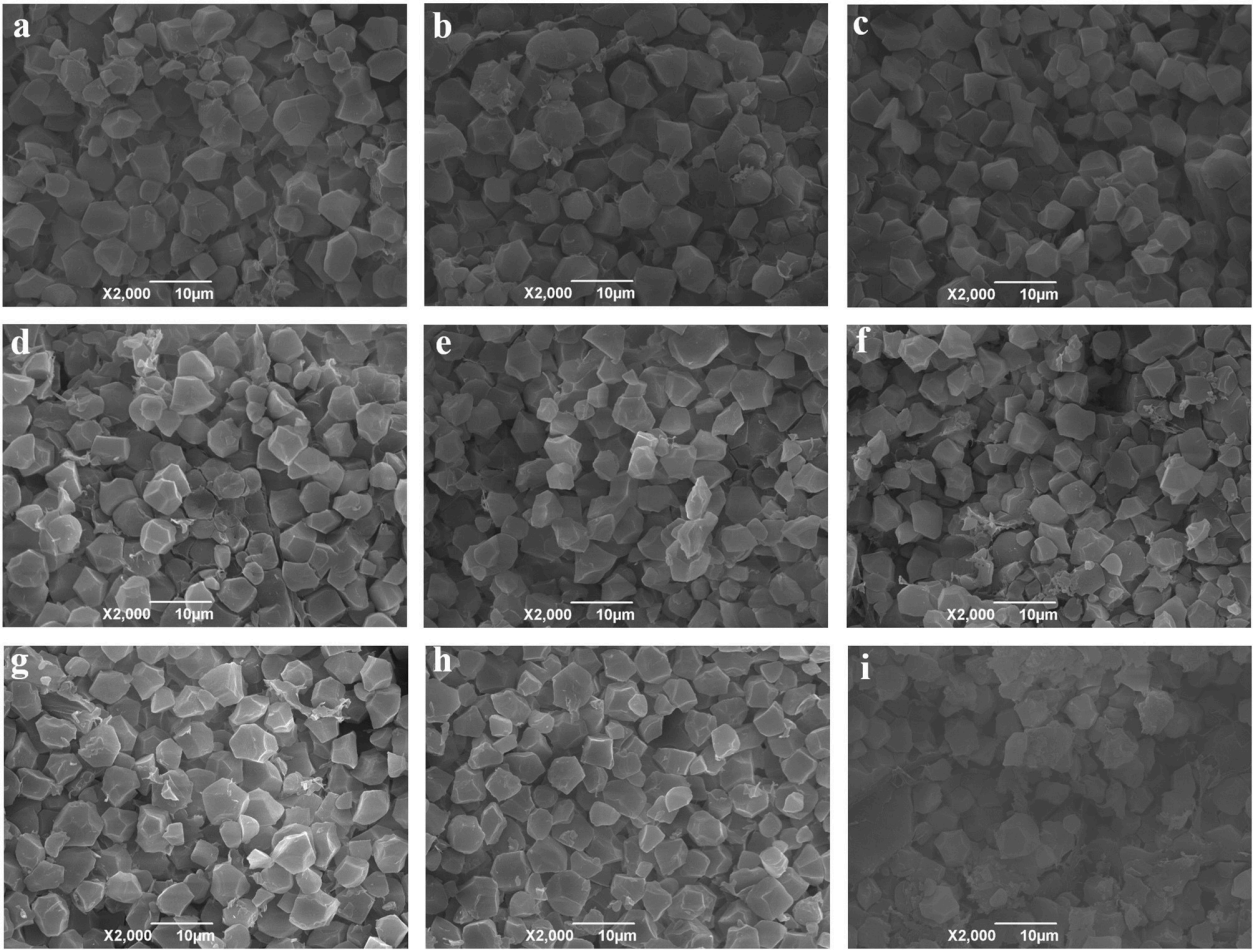
Starch granule in endosperms of hydro-primed rice seeds stored under LT-V, RT-V, RT-A-LH, and RT-A-HH for 0, 30, 60 days. **(a)** 0 DAP control, **(b)** 30 DAP under LT-V, **(c)** 30 DAP under RT-V, **(d)** 30 DAP under RT-A-LH, **(e)** 30 DAP under RT-A-HH, **(f)** 60 DAP under LT-V,**(g)** 60 DAP under RT-V, **(h)** 60 DAP under RT-A-LH, **(i)** 60 DAP under RT-A-HH. DAP, days after priming; LT-V, the primed and non-primed seeds were stored under vacuum, low temperature condition; RT-V, the primed and non-primed seeds were stored under vacuum, room temperature condition; RT-A-LH, the primed and non-primed seeds were stored under room temperature, aerobic and low relative humidity condition; RT-A-HH, the primed and non-primed seeds were stored under room temperature, aerobic and high relative humidity condition.

Structural changes in endosperms of primed rice seeds stored under different conditions and durations are presented in Figure 5. For the un-stored primed rice seeds (0 DAP), the starch was accumulated as compound starch grains and no cavity was observed in the endosperm. No obvious changes were found when the primed rice seeds were stored under LT-V (Figures 5b,f), RT-V (Figures 5c,g) and RT-A-LH (Figures 5c,h) for 30 and 60 days, compared with that of the un-stored seeds (Figure 5a). Nevertheless, storage of primed seeds under RT-A-HH resulted in severely consumption of stored reserves in seed endosperms. When the primed seeds were stored under RT-A-HH for 30 days, the starch grains began to digest into tiny starch granules (Figure 5e). When the storage duration extended to 60 days, the structure of the starch granules were severely destroyed as the surface of the starch grains became rough and irregular, small holes and the cavities between the embryo and endosperm can be observed (Figure 5i).

### Lipid peroxidation

**Figure 6:**
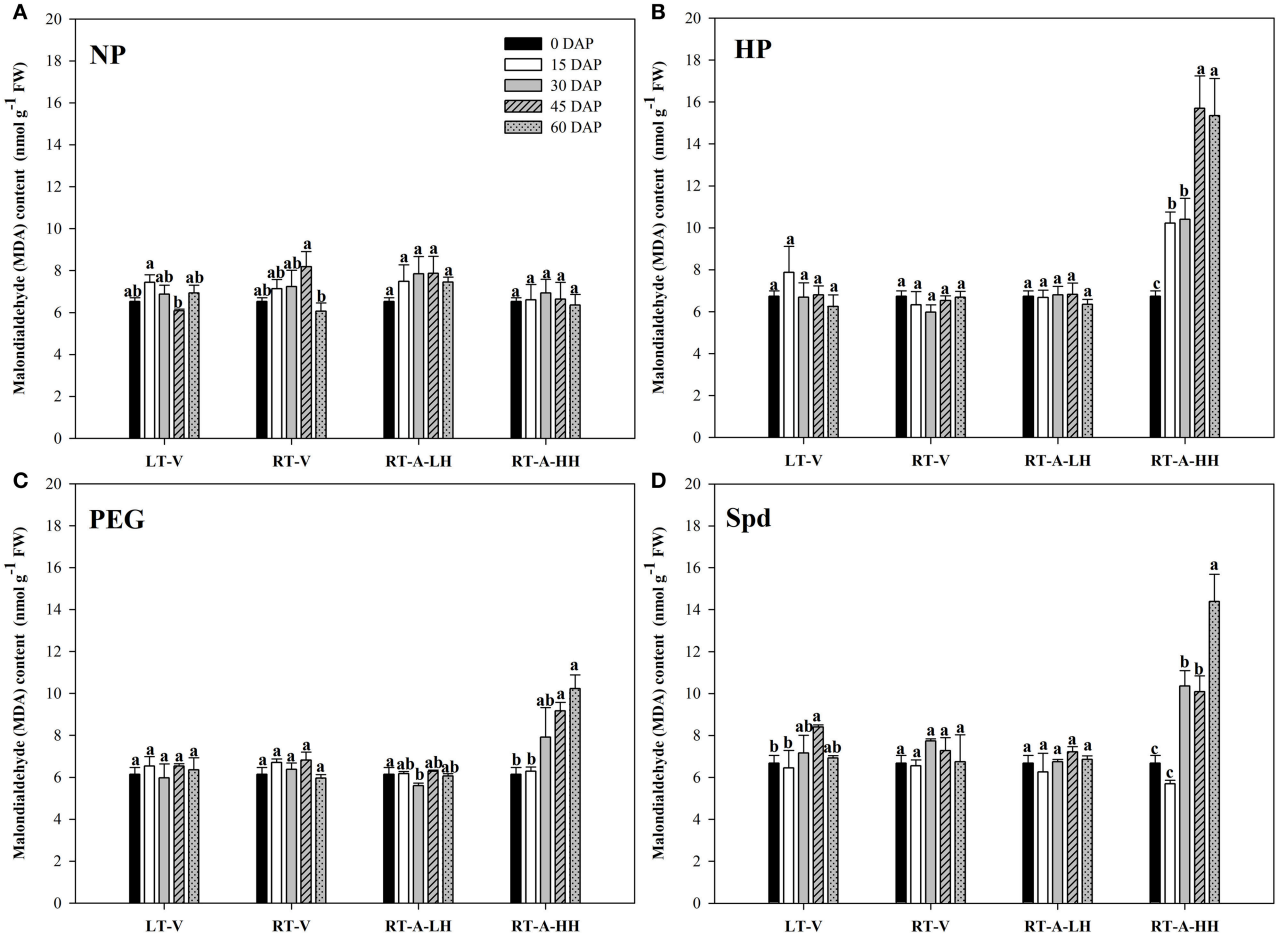
Variations in Malondialdehyde (MDA) content of rice seeds and seedlings in primed and non-primed rice seeds under different storage conditions and storage durations. **(A)** Non-primed control, **(B)** Hydro-priming, **(C)** Osmopriming, **(D)** Spermidine priming. DAS, days after sowing; DAP, days after priming; NP, non-primed control; HP, hydro-priming; PEG, Osmopriming; Spd, spermidine priming; LT-V, the primed and non-primed seeds were stored under vacuum, low temperature condition; RT-V, the primed and non-primed seeds were stored under vacuum, room temperature condition; RT-A-LH, the primed and non-primed seeds were stored under room temperature, aerobic and low relative humidity condition; RT-A-HH, the primed and non-primed seeds were stored under room temperature, aerobic and high relative humidity condition. Error bar above mean indicate stand error (*n* = 4). Different letters denote difference between storage durations of a storage condition at the 5% level according to LSD test.

No significant difference was found in MDA contents when the non-primed rice seeds were stored under different storage conditions and durations (Figure 6). Similar results were also observed for the primed rice seeds stored at LT-V, RT-V and RT-A-LH (Figure 6). Nevertheless, storage of primed rice seeds at RT-A-HH for 15 days significantly increased the MDA contents in rice seeds. Further increase of storage duration brought concomitant increases of MDA contents in primed rice seeds. When averaged across priming treatments, the MDA contents in rice seeds increased by 13.58, 46.63, 78.78, and 104.44%, when stored for 15, 30, 45, and 60 days respectively, as compared with 0 DAP treatment.

### Antioxidant enzymes

**Figure 7:**
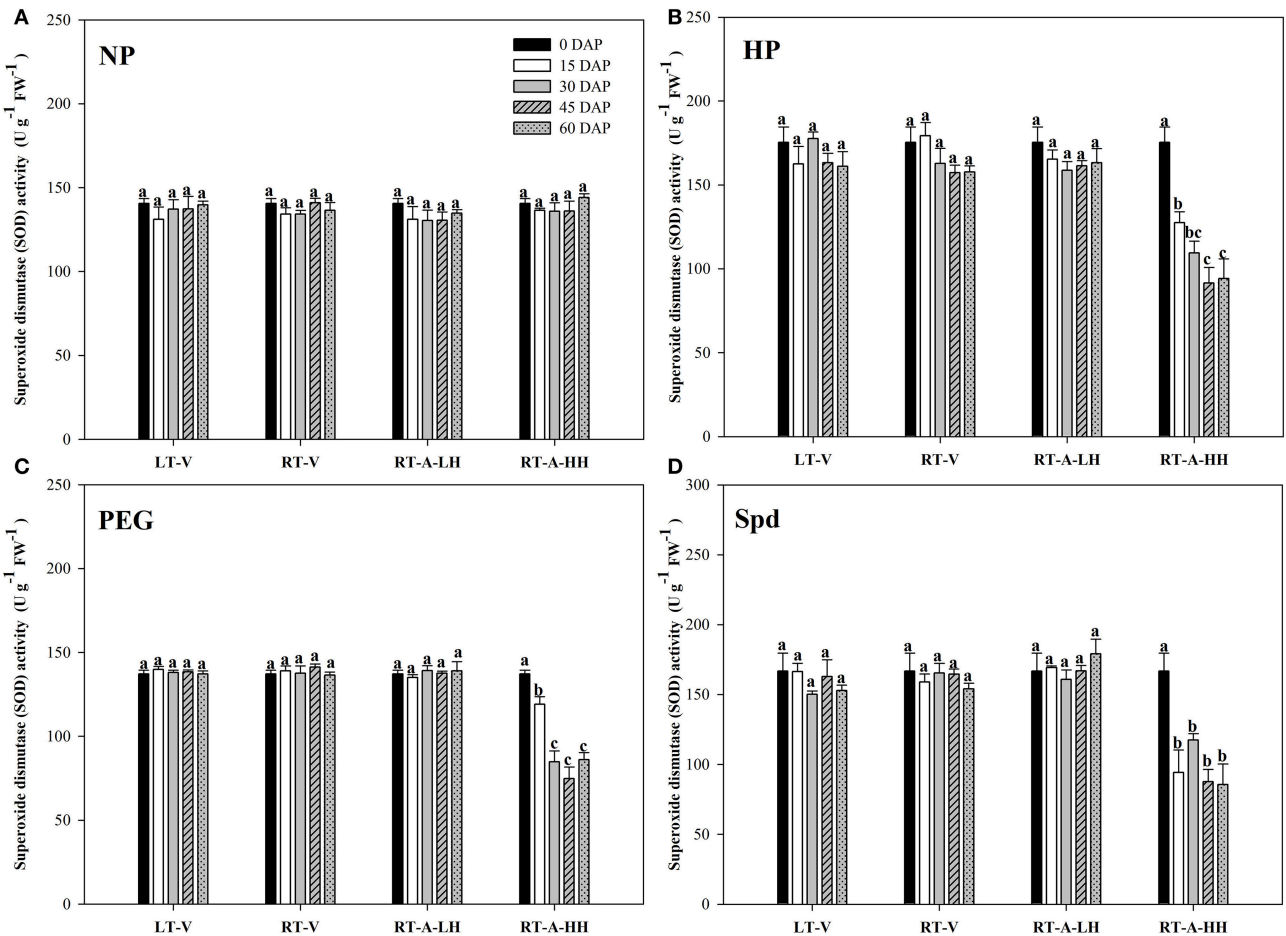
Variations in superoxide dismutase (SOD) activity in primed and non-primed rice seeds under different storage conditions and storage durations. **(A)** Non-primed control, **(B)** Hydro-priming, **(C)** Osmopriming, **(D)** Spermidine priming. DAS, days after sowing; DAP, days after priming; NP, non-primed control; HP, hydro-priming; PEG, Osmopriming; Spd, spermidine priming; LT-V, the primed and non-primed seeds were stored under vacuum, low temperature condition; RT-V, the primed and non-primed seeds were stored under vacuum, room temperature condition; RT-A-LH, the primed and non-primed seeds were stored under room temperature, aerobic and low relative humidity condition; RT-A-HH, the primed and non-primed seeds were stored under room temperature, aerobic and high relative humidity condition. Error bar above mean indicate stand error (*n* = 4). Different letters denote difference between storage durations of a storage condition at the 5% level according to LSD test.

**Figure 8:**
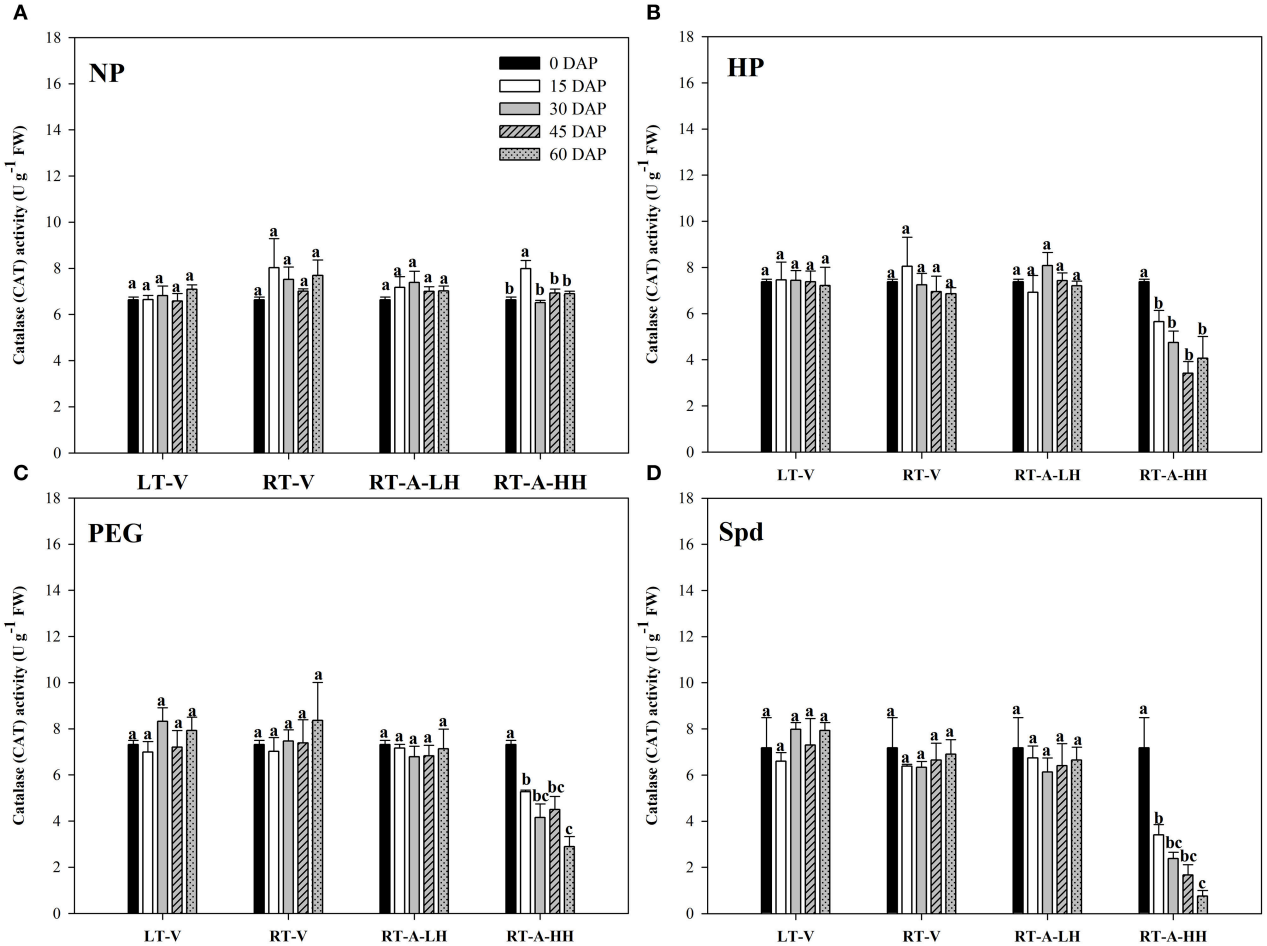
Variations in catalase (CAT) activity in primed and non-primed rice seeds under different storage conditions and storage durations **(A)** Non-primed control, **(B)** Hydro-priming, **(C)** Osmopriming, **(D)** Spermidine priming. DAS, days after sowing; DAP, days after priming; NP, non-primed control; HP, hydro-priming; PEG, Osmopriming; Spd, spermidine priming; LT-V, the primed and non-primed seeds were stored under vacuum, low temperature condition; RT-V, the primed and non-primed seeds were stored under vacuum, room temperature condition; RT-A-LH, the primed and non-primed seeds were stored under room temperature, aerobic and low relative humidity condition; RT-A-HH, the primed and non-primed seeds were stored under room temperature, aerobic and high relative humidity condition. Error bar above mean indicate stand error (*n* = 4). Different letters denote difference between storage durations of a storage condition at the 5% level according to LSD test.

**Figure 9:**
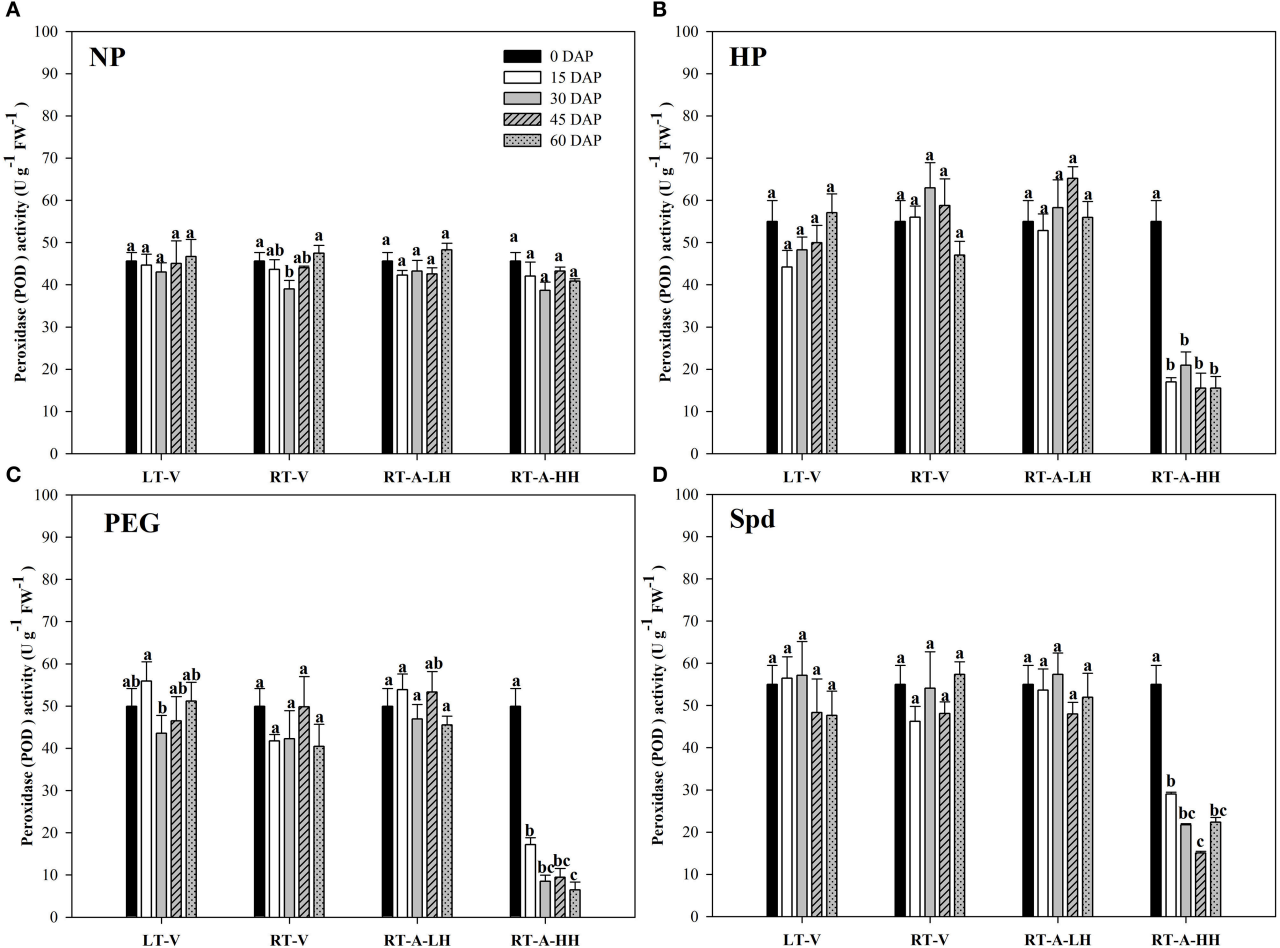
Variations in peroxidase (POD) activity in primed and non-primed rice seeds under different storage conditions and storage durations **(A)** Non-primed control, **(B)** Hydro-priming, **(C)** Osmopriming, **(D)** Spermidine priming. DAS, days after sowing; DAP, days after priming; NP, non-primed control; HP, hydro-priming; PEG, Osmopriming; Spd, spermidine priming; LT-V, the primed and non-primed seeds were stored under vacuum, low temperature condition; RT-V, the primed and non-primed seeds were stored under vacuum, room temperature condition; RT-A-LH, the primed and non-primed seeds were stored under room temperature, aerobic and low relative humidity condition; RT-A-HH, the primed and non-primed seeds were stored under room temperature, aerobic and high relative humidity condition. Error bar above mean indicate stand error (*n* = 4). Different letters denote difference between storage durations of a storage condition at the 5% level according to LSD test.

The activity of antioxidant enzymes (SOD, CAT, POD) in primed and non-primed rice seeds stored under various conditions and durations are presented in Figures 7–9. Consistent with the lipid peroxidation levels, the antioxidant enzymes activity in non-primed rice seeds showed no significant difference among various storage durations and conditions. Similarly, the SOD, CAT, and POD activity of primed seeds stored under LT-V, RT-V, and RT-A-LH for 60 days were not decreased as compared with that of the un-stored control. However, when the primed-seeds were stored under RT-A-HH, all the antioxidants enzymes activities were significantly decreased. When averaged across three priming treatments, the SOD, CAT and POD activity of the primed seeds stored under RT-A-HH for 15 days were decreased by 28.84, 34.44, and 60.48% respectively as compared with that of un-stored control. When the storage duration was extended from 15 to 60 days, the SOD, CAT, and POD activity of the primed rice seeds stored under RT-A-HH were decreased by 34.94–47.00%, 48.44–64.73%, and 67.98–74.94%, respectively, as compared with that of un-stored control.

## Discussion

In the present study, increasing storage temperature did not reduce the viability of primed and non-primed rice seeds stored under vacuum condition within 60 days of storage. In contrast, it has been suggested that the seed deterioration during storage was temperature dependent in brassicaceae species (Mira et al., 2015). The interrelationship between seed moisture content and storage temperature plays more important role in influencing seed viability during storage. Hill et al. (2007) revealed that primed lettuce seeds stored at 9% seed moisture content and 38.8? deteriorated faster than primed seeds stored at 6% seed moisture content at 48.8?. This suggested the more important role of seed moisture content than temperature in influencing seed longevity during storage. In present study, the moisture contents of primed and non-primed seeds were kept under 10% before storage. The viability of primed and unprimed rice seeds were not reduced under RT-V, probably because vacuum treatment enables seeds to keep low moisture content as oxygen and water was isolated from the storage condition. Moreover, the seed respiration might be largely reduced with low seed moisture and the absence of oxygen (Hu et al., 2006). Previously, the beneficial effect of vacuum storage has been observed by several researches. Feng et al. (2017) reported that the moisture content of *P. grandiflorum* seeds did not increase after 10 months of vacuum storage. The beneficial effect of vacuum storage on extending the seed longevity has also been reported in sweet corn seeds (Chiu et al., 2003). Yeh et al. (2005) reported that the longevity of primed bitter gourd seeds were markedly decreased after 12 months storage under ambient oxygen condition, but seed viability was kept under partial vacuum storage condition. Even with high seed moisture content, the deterioration of rice seeds can be delayed for a few weeks at 30°C by vacuum storage (Kaloyereas, 1955). The results of present work suggested that the primed rice seeds can be stored under vacuum condition for 60 days at room temperature if the seed moisture content was below 10%. However, the effect of vacuum storage might be varied among different species, seed moisture contents and storage temperatures. Quantitative evaluation of vacuum storage of primed seeds in response to different seed moisture contents and temperatures needs to be addressed.

At low RH and room temperature, the storage of primed seeds under aerobic environment did not reduce seed longevity during 60 days of storage. As no significant variance was found in germination and seedling growth attributes between RT-V and RT-A-LH. Storage of seeds under aerobic condition has been reported to result in several changes caused by respiration, such as the depletion of food reserves (proteins and sugar contents), which were considered to be responsible for seed aging (Calucci et al., 2004). Schwember and Bradford (2011) reported that storage of primed lettuce in aerobic condition seeds accelerated the seed deterioration as compared with that of anaerobic condition during 2 years of storage. Ellis et al. (2008) concluded that the effect of oxygen on seed deterioration increased as seed moisture content decreased. However, such negative effect was not observed in present study, this is probably because that the 60 days of storage was too short to reach the threshold point of seed deterioration. Besides, the oxygen concentration in the storage condition might not reach the point to trigger series of physiological metabolisms that could deplete longevity of primed and unprimed rice seeds. Further studies to examine the role of oxygen in influencing the viability of primed rice seeds during storage are needed.

Compared with RT-A-LH, increasing RH (RT-A-HH) significantly decreased germination and seedling growth attributes of primed rice seeds, which suggested that storage of primed rice seeds at high RH was mainly responsible for deterioration of primed rice seeds in present study. These results were consistent with several researches that reported high RH, high temperature reduced the seed longevity during storage (Powell and Matthews, 1977; Suma et al., 2013). High RH would directly lead to an increase in seed moisture content, which is detrimical for the viability of seeds during storage (Abba and Lovato, 1999). High seed moisture content along with high temperature and ambient oxygen would trigger series of metabolic changes, such as the accumulation of the reactive oxygen species, loss of membrane integrity, increase in respiration and the consumption of storage reserves, and thus lead to seed deterioration (McDonald, 1999; Liu et al., 2016). In contrary with primed rice seeds, the longevity of non-primed rice seeds stored under RT-A-HH did not reduce, suggesting that that primed rice seeds are more sensitive to high RH and high temperature than non-primed seeds. Previously, Hacisalihoglu et al. (1999) have also observed that the germination potential and percentage of primed lettuce seeds was significantly decreased exhibited when stored under mild storage conditions (45? at 50% relative humidity). It is might because primed seeds are easier to suffer from oxidation or damage than non-primed seeds during storage, which resulted in faster seed deterioration (Hussain et al., 2015). In summary, present study suggested that with low relative air humidity, the primed rice seeds could be aerobically stored at room temperature for 60 days, while increasing RH would drastically lose the viability of primed rice seeds.

In the present study, the reduction in starch metabolism and the consumption of stored reserves in the endosperms might be responsible for the viability loss of primed rice seeds stored under RT-A-HH. Starch metabolism is the major driving force for rice seed germination and could support plants to grow faster under various environment (Murata et al., 1968). Reduced starch metabolism in primed seedlings were strongly correlated with the decreased germination percentage and seedling growth attributes. The restriction of starch metabolism in primed rice seed during storage might be caused by the accumulation of reactive oxygen species. Storage of primed seeds under unfavorable condition would impair the antioxidative systems (Chang and Sung, 1998), lead to the damage of cell structures (Kaewnaree et al., 2011), break the metabolism balance and ultimately hampered the synthesize of associated amylase proteins. The consumption of starch reserves in the endosperms might be another reason for the reduced starch metabolism of primed rice seeds after storage. However, it has been suggested that even with the depletion of food reserves, seeds still have ample energy to support the germination process (Justice and Bass, 1978). The pre-consumption of starch reserves in primed seeds might result in a breakdown of food transport system (Harrington, 1960). Besides, Oxley (1948) suggested that exhaustion of an unnamed organic compound resulted in loss of seed viability during storage. However, the mechanisms associated with the deterioration of primed seeds during storage are not fully understood and need to be studied further.

The deterioration of primed rice seeds stored under RT-A-HH was closely related to the increased lipid peroxidation level (reflected by the MDA content) in the present study (Figure 6). This results were consistent with previous research showing that lipid peroxidation was one of the major causes of seed aging during storage (Maskri et al., 2003; Sung and Jeng, 2010; Veselovsky and Veselova, 2012). Our present study also indicated that the primed rice seeds were much easier to suffer from lipid peroxidation damage than non-primed rice seeds during storage (Figure 6). This might be because that priming treatments could activate several metabolic events in rice seeds, which accelerates the ROS accumulation and thus leads to the increased lipid peroxidation levels during storage. The decreased activity of POD, CAT and SOD were also responsible for the increased lipid peroxidation levels in primed rice seeds stored at RT-A-HH (Figures 7–9). The decreases of antioxidant enzymes activity severely hampered ROS scavenge and accelerated lipid peroxidation. In summary, our present study suggested that the increased lipid peroxidation levels and decreased antioxidant enzyme activities were associated with the deterioration of primed rice seeds stored at RT-A-HH. However, the regulation mechanisms associated with the ROS accumulation and scavenge in primed rice seeds under various storage conditions and durations need to be explored.

In addition to starch metabolism, antioxidant enzymes activity and lipid peroxidation, others have also been suggested to be associated with seed deterioration during storage. Petla et al. (2016) demonstrated the important role of PROTEIN L-ISOASPARTYL O-METHYLTRANSFERASE (PIMT) in regulating seed longevity, and suggested that PIMT repaired antioxidative enzymes and proteins which restrict lipid peroxidation and thus increased seed longevity (Petla et al., 2016). Yin et al. (2017) indicated that protein oxidation might be responsible for seed aging. Eisvand et al. (2010) reported that hormone priming could alleviate the damaging effect of seed aging. Kranner et al. (2011) suggested that DNA fragmentation and loss of RNA integrity were strongly linked with seed aging. In addition, the proteomic and transcriptomic analysis in primed and non-primed rice seeds under various different storage conditions and storage durations will be examined in our further work.

Almost similar responses of three priming treatments in present studies to varying seeds storage conditions and durations suggests the possibility for applicability of these findings to wide range of priming agents in rice crop. However, seed storage potential may still differ among different cultivars and species. Further studies should concentrate on screening and breeding cultivars with high storage potential.

## Conclusion

Our study conclusively revealed that storage of primed seeds under high RH condition beyond 15 days is deteriorative for germination and growth of rice, and such damaging effects were related with reduced starch metabolism, consumption of starch reserves in rice endosperm, increased lipid peroxidation levels and decreased antioxidant enzyme activities. However, if primed seeds were stored at vacuum or low RH or low temperature conditions, there will be no negative effect on seed viability within 60 days of storage. These findings will have practical implications for the application and promotion of seed priming.

## Author contributions

WW and LN: initiated and designed the research; WW and AH: performed the experiments, WW and LN: analyzed the data and wrote the manuscript, SP, JH, and KC revised and edited the manuscript and also provided advice on the experiments.

### Conflict of interest statement

The authors declare that the research was conducted in the absence of any commercial or financial relationships that could be construed as a potential conflict of interest.
